# Surface Characteristics, Fluoride Release and Bond Strength Evaluation of Four Orthodontic Adhesives

**DOI:** 10.3390/ma14133578

**Published:** 2021-06-26

**Authors:** Mihaela Pastrav, Andrea Maria Chisnoiu, Ovidiu Pastrav, Codruta Sarosi, Doina Pordan, Ioan Petean, Alexandrina Muntean, Marioara Moldovan, Radu Marcel Chisnoiu

**Affiliations:** 1 Department of Orthodontics, “Iuliu Hațieganu” University of Medicine and Pharmacy, 31 Avram Iancu Street, 400117 Cluj-Napoca, Romania; mihaela.pastrav@umfcluj.ro; 2 Department of Prosthodontics, “Iuliu Hațieganu” University of Medicine and Pharmacy, 32 Clinicilor Street, 400006 Cluj-Napoca, Romania; 3 Department of Odontology, Endodontics and Oral Pathology, “Iuliu Hațieganu” University of Medicine and Pharmacy, 33 Motilor Street, 400001 Cluj-Napoca, Romania; ovidiu.pastrav@umfcluj.ro (O.P.); marcel.chisnoiu@umfcluj.ro (R.M.C.); 4 “Raluca Ripan” Institute of Research in Chemistry, “Babes Bolyai” University, 30 Fantanele Street, 400294 Cluj-Napoca, Romania; doina.prodan@ubbcluj.ro (D.P.); marioara.moldovan@ubbcluj.ro (M.M.); 5 Faculty of Chemistry and Chemical Engineering, “Babes-Bolyai” University, 11 Arany Janos Street, 400084 Cluj-Napoca, Romania; ioan.petean@ubbcluj.ro; 6 Department of Pedodontics, “Iuliu Hatieganu” University of Medicine and Pharmacy, 31 Avram Iancu Street, 400117 Cluj-Napoca, Romania; alexandrina.muntean@umfcluj.ro

**Keywords:** metallic bracket, orthodontic adhesive, fluor release, share bond strength, roughness

## Abstract

Orthodontic adhesives have similar properties in terms of fluoride release, roughness, shear bond strength or cement debris for specific clinical conditions. Three commercial consecrated orthodontic adhesives (Opal Seal^®^, Blugloo^®^, Light Bond^®^) were compared with an experimental orthodontic material (C1). Brackets were bonded to enamel using a self-etch technique followed by adhesive application and then de-bonded 60 days later. Share bond strength evaluation, scanning electron microscopy, atomic force microscopy and fluoride release analysis were performed. The highest amount of daily and cumulative fluoride release was obtained for the experimental material, while the lowest value was observed for Opal Seal^®^. The materials evaluated in the current study presented adequate shear bond strength, with the experimental material having a mean value higher than Opal Seal and Blugloo. The atomic force microscopy measurements indicated that the smoothest initial sample is Opal Seal^®^ followed by Light Bond^®^. Scanning electron microscopy evaluation indicated different aspects of cement debris on the enamel and/or bracket surface, according to the type of adhesive. The experimental material C1 presented adequate properties in terms of shear bond strength, fluoride release, roughness and enamel characteristics after de-bonding, compared to the commercial materials. Under these circumstances, it can be considered for clinical testing.

## 1. Introduction

Multi-bracket treatment still represents the most widely used type of treatment among orthodontic applications, especially during childhood and adolescence when it is the treatment of choice [1]. It has advantages, but also risks, most frequently being encountered incidents such as bracket detachment, or development of gingival inflammation, increased susceptibility to caries, decalcifications and white spot lesions [2,3].

White spot lesions’ occurrence has been reported to vary from 2% to 97%, while in patients without orthodontic treatment it is between 11% and 24% [4,5]. They are caused by enamel demineralization due to bacterial plaque deposits and carbohydrate metabolism with acidic release. Plaque retention is accentuated by bands, brackets and arches used during multi bracket treatment. In this context, it is very important to use and improve a remineralization protocol, which reduces acid production and increases enamel resistance to demineralization [6]. Previous studies report that fluoride-containing restorative materials maintain the fluoride concentration in the oral saliva at 0.03 ppm for one year and can inhibit demineralization up to 7 mm from the edge of restoration [7,8]. An amount of 200–300 μg/cm^2^ of fluor released per month is considered sufficient to assure demineralization protection [7].

Another major concern during multi bracket orthodontic treatment is represented by bracket detachment. This situation might be critical in some clinical situations as it can influence the overall success of the treatment. Previous research has reported various incidence rates of bracket detachment, from 28.3% to relatively low incidence values of 0.6–9.6% [9]. Other studies have also compared several techniques and materials for orthodontic bonding [10,11,12].

The development of recent technology in materials and adhesion techniques makes orthodontic bonding more predictable and efficient. Nowadays, indirect and direct bonding techniques are available. The indirect bonding technique requires laboratory intervention, so it is generally avoided by orthodontists, but it has a higher degree of precision, predictable results and shorter appointment time [13].

Various materials are available for bracket bonding, from conventional cements to dual cure resin, or glass ionomer cements. Resin fluoride-containing cements were developed in order to increase fluoride release and shear bond strength [14].

This study aims at evaluating the fluor release and shear bond strength of four orthodontic cements (three consecrated commercial adhesives (Opal Seal (Opal Orthodontics, South Jordan, UT, USA), Blugloo (Ormco Corporation, Glendora, CA, USA), Light Bond (Reliance Ortho Prod. Inc., Itasca, IL, USA)) and one experimental (C1)) as well as observing, by means of scanning electron microscopy (SEM), the dental tissue characteristics after bracket detachment. Using atomic force microscopy (AFM), roughness measurements were performed initially and after fluoride release evaluation.

The null hypothesis of the current study was that no statistically significant difference could be found between the new experimental material and three consecrated orthodontic adhesives.

## 2. Materials and Methods

Forty decay-free permanent premolars were included in the study. The teeth were extracted on orthodontic indication. All subjects gave their informed consent for inclusion before they participated in the study. The study was conducted in accordance with the Declaration of Helsinki, and the protocol was approved by the Ethics Committee of University of Medicine and Pharmacy Cluj-Napoca, approval number 180/06.05.2020.

Four types of orthodontic adhesives (Blugloo, Light Bond, Opal Seal and experimental material (C1) were used (Table 1). All brackets were bonded to enamel using self-etch technique followed by adhesive application.

The teeth were suspended in 45 ml double-distilled water/5 mL TISAB III buffer (Total ionic strength adjustment buffer, concentrated solution, HI 4010-06, Hanna Instruments, Woonsocket, RI, USA) at 37 °C.

The metal bracket bonding procedure was conducted according to the manufacturer’s indications and kept in artificial saliva for 24 h. Artificial saliva was obtained in the laboratory, simulating the salt composition of saliva, according to the following recipe: 0.4 g/L KCl, 0.4 g/L NaCl, 0.69 g/L NaH2PO4 H2O, 0.005 g/l Na2S 9H2O, 0.795 g/L CaCl2 2H2O 1.0 g/L CO(NH2)2, aqueous solution [15].

The buccal surface was etched for 30 s with 37 percent phosphoric acid at room temperature, rinsed for 15 s with water, and air dried until a white, chalky surface appeared. Subsequently, after application of the Transbond XT primer, a light cure orthodontic adhesive (Transbond XT, 3M Unitek, Monrovia, CA, USA) was used to bond the stainless-steel brackets to the tooth surface. All brackets were light cured using a light-curing unit (Woodpecker LED Curing light, Guilin Woodpecker Medical Instrument Co., Ltd.; Guangxi, China, absorbs light in the 400–500 nm, I ≈ 600 mW cm^−2^, light guide diameter 8 mm) for 40 s (10 s for mesial, distal, gingival and occlusal sides). Excess adhesive was removed using an explorer before light curing.

**Figure 1:**
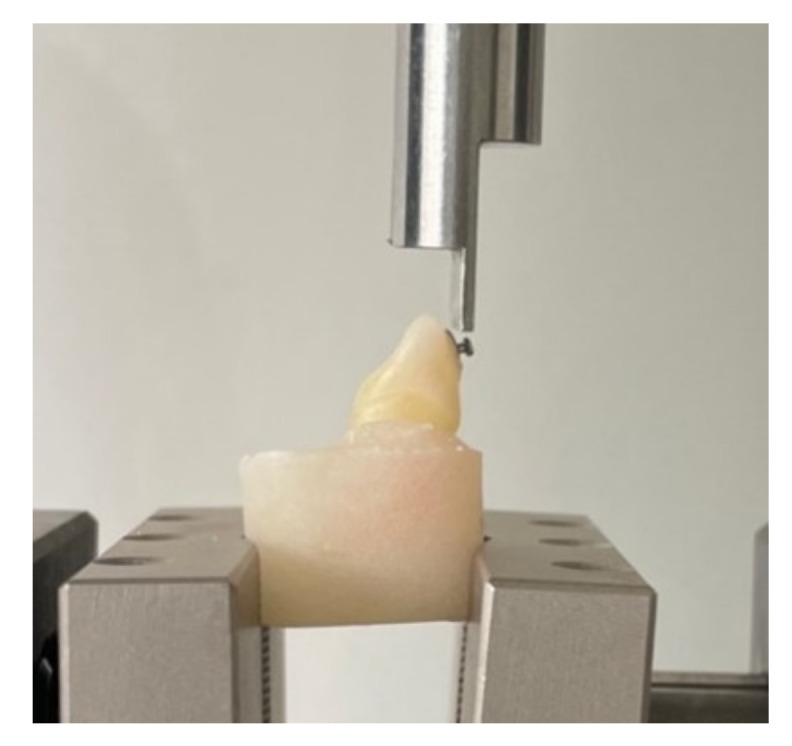
Bracket debonding procedure using Loyd Universal Testing machine.

The brackets were debonded 60 days after the initial moment of the experiment. Bond strength force was evaluated using the Loyd Universal Testing machine (LF Plus, LLOYD, Instrument, Ametek Inc., West Sussex, UK) (Figure 1). A sharp blade was used to apply an ocluso gingival force at the bracket-adhesive interface, with a speed of 1 mm/min. The values of the bond strength force were measured in MPa, using NEXYGEN Plus Materials Testing Software (version 3.0, Lloyd Instruments Ltd. Steyning Way, Bognor Regis, West Sussex, UK).

For fluoride ions release and AFM analysis, 20 disk-type material samples were realized using a Teflon mold (5 samples/ material) with the dimensions: diameter d = 15 ± 1 mm and thickness h = 1 mm. The light-curing was carried out with the same light-curing unit for 180 s (9 points on both sides).

### 2.1. SEM Analysis

All teeth samples prepared for SEM analysis were immersed in 2.5% sodium hypochlorite for 1 min, 17% EDTA for 2 min and finally three times distilled water for 2 min (R. Espinar-type ultrasound bath (RAYPA Company, Terrassa, Spain). The teeth samples were examined using a gold-rich substrate-QUANTA 133 (FEI Company, Hillsboro, OR, USA). Several image magnification units were used and recordings were made on both the bracket surface and the tooth. The main purpose of the SEM evaluation is to observe the possible morphological changes of the enamel as well as to establish correlations between the fracture area patterns depending on the materials used.

### 2.2. Determination of Fluoride Ions Release

For fluoride ions release, daily measurements were performed for the first 3 days and then weekly until the end of the 60 days, according to previous studies [15,16]. After each measurement, the material samples was placed in the same storage container (made of polyethylene), and kept in a thermostatic bath at 37 °C. Fluoride ion analysis was performed using a selective fluoride ion electrode (Combination Fluoride Electrode HI 4110 filled with HI 7075 electrolyte for the reference electrode, Hanna instruments).

The electrode was pre-calibrated, using a series of calibration solutions of concentrations between 10^−6^ and 10^−2^ mol/L starting from a basic solution of 1M NaF (Merck KGaA, Darmstadt, Germany). Calibration solutions were used to plot the calibration curve. All measurements, both for the calibration solutions and for the solutions to be investigated, were performed in 50 ml of double distilled water/TISAB III (Hanna Instruments INC, Woonsocket, RI, USA) buffer (45:5) at 37 °C (±2). The release of fluoride ions was expressed in ppm.

### 2.3. AFM Analysis

The material samples were investigated by AFM in the complete dry state, in natural atmosphere at 20 °C in tapping mode. A JEOL JSPM 4210 Scanning Probe Microscope, produced by JEOL, Tokyo, Japan was used along with the cantilevers of NSC 15 type produced by MicroMasch, Talinn, Estonia. The cantilever resonant frequency is of about 325 kHz and the force constant of 40 N/m. The images were scanned at area of 5 µm × 5 µm for at least 5 different macroscopic areas. All images were processed in standard manner using Jeol WIN SPM 2.0 processing soft (JEOL Ltd., Akishima, Tokyo, Japan), average roughness (Ra) and root mean squared roughness (Rq) being measured for each image.

### 2.4. Statistical Analysis

All data were collected and statistically analyzed using SPSS Statistics (ver. 20.0, Chicago, IL, USA). After checking the normality and equality of variance of the data, comparison of shear bond strength values (MPa) was performed using a two-way ANOVA test and a Student–Newman–Keuls post-hoc test. The two-paired Wilcoxon tests was used to compare the results of roughness for the experimental material versus the three consecrated materials. *p* values < 0.05 were considered statistically significant.

## 3. Results

**Figure 2:**
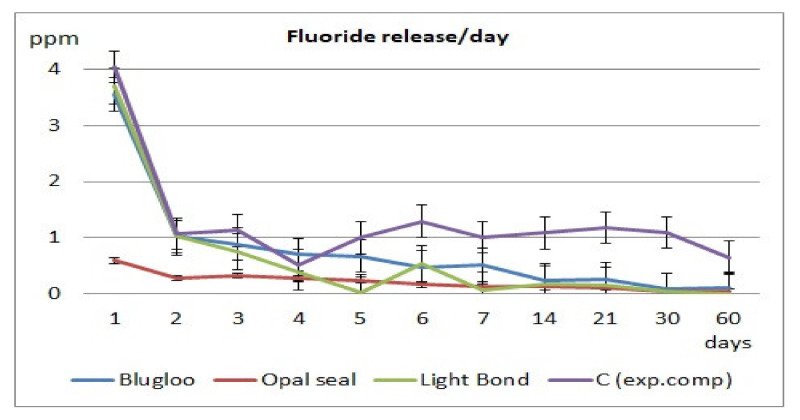
Daily fluoride release for the tested materials.

**Figure 3:**
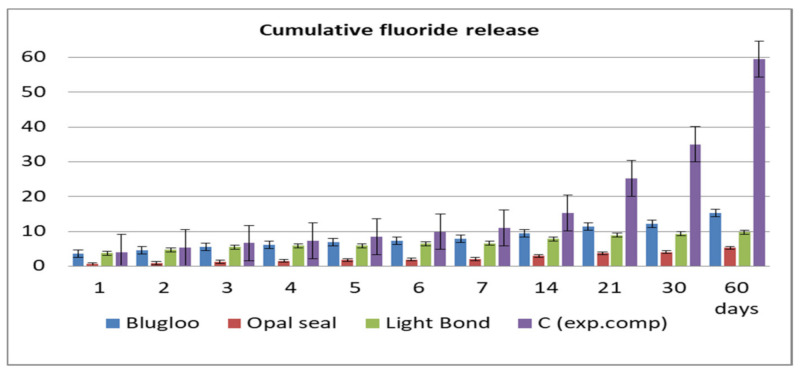
Cumulative fluoride release of the four materials.

Fluoride release values per day and cumulative during the 60 days of testing are displayed in Figure 2 and Figure 3.

Differences in values of shear bond strength for the tested materials were statistically validated. The highest mean value was observed in case of Light Bond, while the lowest was identified for Opal Seal (Table 2).

The topographic AFM images that resulted for the initial material samples are presented in Figure 1 and the roughness values are presented in Table 3. Each sample has a different topography, which is in a close relation to the micro and nanostructure within the composites.

**Figure 4:**
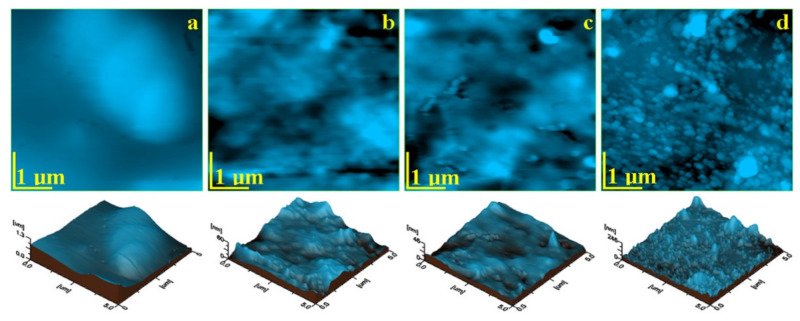
AFM topographic images of the initial samples’ surfaces: (**a**) Blugloo, (**b**) Light Bond, (**c**) Opal Seal, and (**d**) Experimental material C1. Three-dimensional images of each topographic image are given below.

The AFM image obtained for Blugloo initial material sample, Figure 4a, shows a very compact surface, which indicates an optimal embedding of the filler into the polymer matrix. None of the filling components are visible on the sample surface. Despite the local smoothness of the surface, some micro-structural irregularities occur as some blunted hills lead to relatively increased values of Ra and Rq roughness. These irregularities are more visible in the three-dimensional image.

The topographic image for Light Bond in Figure 4b evidenced submicron mineral particles, with diameters of around 250 nm, very well embedded into the polymer, which forms a compact microstructure. Fused silica submicron particles are very well dispersed into the polymer matrix, which leads to a good smoothness of the surface and to relatively low roughness.

The topographic image for C1 (Figure 4d) shows a very compact surface, which contains rounded nanoparticles that are very well embedded into the polymer. Their diameter ranges from 40 to 60 nm, but a pronounced clusterization tendency occurs. Then, the filler nanoparticles form some regular clusters of about 120–180 nm, with very few having diameters of 600 nm. This cluster-forming tendency led to relatively increased values of Ra and Rq.

**Figure 5:**
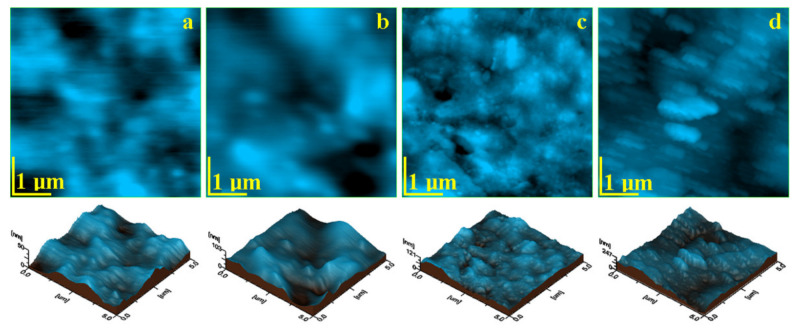
AFM topographic images of the samples surfaces after 60 days of exposure: (**a**) Blugloo, (**b**) Light Bond, (**c**) Opal Seal, and (**d**) Experimental material C1.

It is expected that the prolonged exposure for 60 days to the wet environment (e.g., water with TISAB III) will affect the surface of each sample in a particular manner. The surface alteration was observed by the AFM topographic images, shown in Figure 5, and the measured roughness parameters Ra and Rq were centralized in Table 4.

Opal seal is affected by erosion after 60 days of exposure in a similar manner to the one observed for Light Bond. The outermost polymer layer is more affected by prolonged contact with the wet environment as compared to the mineral components, which are more visible on the surface, Figure 5c. This suggests a significant increase in the roughness value (see Table 4). The nano filler particles become visible in the surface with a rounded shape and a diameter of about 60 nm that surrounds the glassionomer particles, which feature a diameter of about 300 nm.

**Figure 6:**
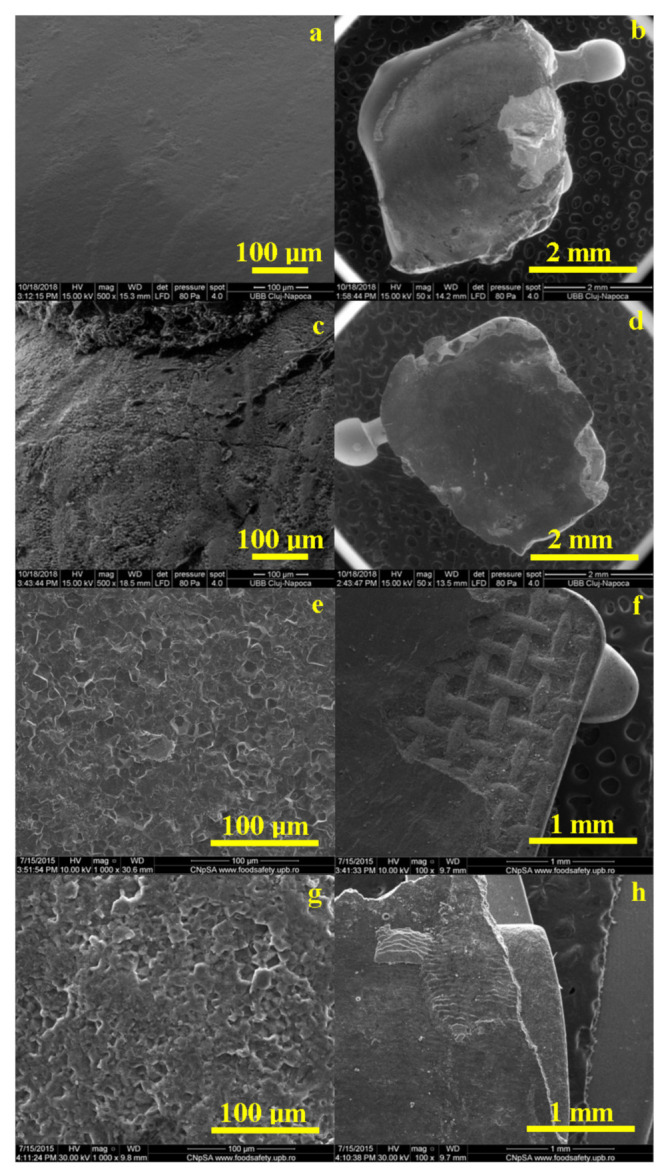
Representative SEM images of the enamel and bracket surface after debonding for all tested orthodontic adhesives: Blugloo (**a**,**b**); Light Bond (**c**,**d**); Opal Seal (**e**,**f**); experimental material C1 (**g**,**h**).

The images in Figure 6 show some defects, which are most likely caused by corrosion in artificial saliva or by various effects combined with separate acid etching. It is observed that, in the case of all materials, the cement remained, for the most part, on the surface of the bracket, the desired situation in the case of these treatments. The largest amount of orthodontic adhesive remanent on the tooth is Opal Seal (Figure 6f).

All cements have a homogeneous structure. The surface of the enamel is affected by the acid etching treatment, with the most pronounced effects observed in Figure 6e,g.

## 4. Discussion

At the present time, various adhesives are available for orthodontic bonding. In the current study, four orthodontic adhesives were analyzed and compared in terms of shear bond strength, roughness, fluoride release and surface characteristics after debonding [17].

Opal Seal is a sealer based on 38% mineral filler including glass ionomer particles and nanofillers that are well dispersed into the polymer matrix. The current study indicates that Opal Seal had the lowest initial roughness, increased bond strength and the lowest fluoride release. These results are similar to those obtained by Kolstad et al. [18].

Experimental material C1 is based on nano structural filler in an amount of 30% of the overall polymer matrix. Although, in the case of experimental cement samples, the roughness had the highest values both initially and after debonding in experimental samples, and the lowest bond strength was identified, the fluoride release values were the highest.

Light Bond is also a composite sealer which contains fused silica as a filler comprising about 50% of the polymer-based matrix. The presence of a large amount of fused silica affects the surface morphology. The Light Bond group showed better protection of the enamel surface than other groups, as indicated from the mean values of roughness. The moderately wide and shallow perikymata grooves, with reduced surface roughness, indicated that there was a loss of the outer few micrometres of the enamel surface due to the softening of the enamel, resulting in localized areas of destruction. This result is similar to previous reported findings [19].

According to Reynolds, shear bond strength in the range of 5.9–7.8 Mpa, to resist masticatory force, is clinically favorable and minimizes enamel fracture. Bond strength that is higher than 14 MPa can cause enamel cracks on the tooth surface during debonding [20]. The materials evaluated in the current study presented adequate shear bond strength, with the experimental material having a higher mean value than the average. 

Shear bond strength depends on several factors, including the size and design of the bracket base, the thickness and type of adhesive, bonding technique, type of bracket, and experience level of the clinician. Light-cured and self-cured conventional composites for bracket bonding exhibit a lack of color contrast with the enamel, which may result in the accumulation of resin remnants on the enamel surface after bracket debonding and polishing [21].

When performing the mechanical test, the failure recorded at the adhesive interface was often a mixed one, situated at the cement–enamel junction for the majority of the samples.

The factors that influence the adhesive debris placement after debonding are the bracket base characteristics, which in the case of metallic brackets increase the contact surface, the mechanical retention and the degree of polymerization of the adhesive under the metal bracket.

The use of different materials as well as the etching technique determined the development of various signs of wear, which were observed during SEM evaluation both on the surface of the tooth and on the bracket. Gaps and cracks of different sizes were observed, probably initiated by the shear forces present at the bracket/cement interface. In the current study, SEM images were used to gain a better understanding of how the enamel surfaces changed after the shear bond test. However, SEM requires subjective inspection, and cannot be used for comparative assessments alone [22].

The AFM measurements indicate that the smoothest initial sample is Opal Seal followed by Light Bond. The roughest samples are Blugloo followed by the experimental material C1. The surface roughness is clearly influenced by the filler particles’ positioning on the polymer matrix microstructure.

The topography of the initial state of the investigated materials depends on the filler type and its bonding to the polymer matrix. The filler dispersion on the matrix is also an important factor. Filler in Blugloo and Experimental material C1 presents a significant tendency towards cluster formation (blunted hills in Blugloo and open clusters on the surface of C1) which leads to increased roughness values. Samples with a better filler dispersion (Light Bond and Opal Seal) form smoother surfaces.

The topography after 60 days of exposure reveals that all samples were affected by erosion. The clusters in Blugloo and C1 are very affected by the erosion, which causes significant reductions in the surface roughness. Light Bond and Opal Seal samples are affected by the mineral loss in their surface, which causes a small increase in the roughness. Despite the observed surface decay, the cohesion of the bulk material is not affected in depth by wet erosion, presenting a good overall stability in moist environments.

Blugloo presents significant changes of the surface morphology after 60 days of exposure. The top layer of the polymer matrix is affected by wet erosion, which causes it to become thinner. The polymer outermost layer is so thin than the filler particles become visible in the topographic image. They have rounded shapes and diameters of around 250 nm. The surface becomes smoother than in the initial state. The changes in topography provide important clues about the erosion mechanism. The blunted hills are totally eroded, becoming areas on the surface where the filler particles are more visible. This indicates that the blunted hills observed in the initial samples are more sensitive to the wet erosion, perhaps due to a local higher accumulation level of filler particle content.

The mineral loss in the surface of Light Bond sample after 60 days of exposure is observed in the topographic image. The filler particles in the surface were subjected to prolonged contact with the wet environment, which implies weakening of their cohesion to the polymer matrix. A significant number of them were washed away from the surface, with only a few remaining visible in the topographic image as rounded particles of 250–300 nm diameter. This affects the surface by enhancing some local depressions (initially well filled with mineral material). It is sustained by the slow increasing of the roughness values.

The surface of experimental material C1 is very affected by the prolonged exposure to the moist environment. The nano filler clusters in the surface are sensitive to the water content of T.S.A.B, which weakens their internal cohesion and leads to significant mineral loss. Thus, the clusters change their shape, becoming prolonged under contact with the cantilever during scanning. It proves their increased friability, allowing the observation of some nanoparticles of about 40–60 nm, which are dragged from clusters in the scan direction. Therefore, the clusters with 120–180 nm in the initial stage become elongated (ellipsoidal like), maintaining their short diameter at 120–180 nm, and the prolonged diameter is situated at around 700 nm. The morphology changes within the C1 surface lead to significant decreases in roughness.

The highest amount of fluoride release was obtained in the case of the experimental cement both in cumulative and per day measurements, followed by Blugloo samples, while the lowest fluoride release was identified in the case of Opal Seal samples. However, all samples had a sufficient fluoride release to ensure a remineralization [23].

As shown on the cumulative and daily fluoride release diagrams, an initial burst is observed, probably due to loosely bound fluoride [24]. Usually, after 7 days, a stabilization phase is observed in fluoride release [25]. The same pattern has been observed in the current study.

The null hypothesis has been rejected, since the experimental material presented significantly improved share bond strength compared to Opal Seal and Blugloo, and higher roughness, both initially and after 60 days, compared to Light Bond and Opal Seal. However, further investigation is necessary, since for clinical utilization, biological and cytotoxicity tests are necessary.

As materials that are in close contact with biological tissues (i.e., teeth and oral soft tissues), either directly or indirectly, biocompatibility is one of the most critical requirements for dental adhesives [26]. It has been demonstrated that components of adhesives and restorative resins such as HEMA (2-hydroxyethyl methacrylate) and TEGDMA (triethylene glycol dimethacrylate), respectively, can diffuse through the dentinal tubules and reach the pulp tissue in concentrations considered toxic to the pulp cells. Investigations on their biocompatibility showed that 90% of residual TEGDMA and HEMA monomers were released within the first 24 hrs. Released monomers could spread through dentine with the risk of hypersensitization and cytotoxicity [27].

The current study has several limitations including the duration of the study, especially for shear bond strength evaluation, with longer studies, using various environmental conditions, being necessary. A clinical comparison of the four sealants is also required to properly evaluate their demineralization efficacy and bonding durability. In general, in vitro experimental results can never succeed completely compared to those obtained in clinical situations, since application-sensitive substrates and the complexity of their interactions are subject to error [28]. However, in vitro experiments do provide valuable information for in vivo usage and are of significant value for current clinical practice.

## 5. Conclusions

The experimental material C1 presented adequate characteristics in terms of shear bond strength, fluoride release, roughness and enamel characteristics after debonding, compared to the commercial materials. Further clinical investigation is necessary to obtain confirmation of these promising results.

## Figures and Tables

**Table 1:** Orthodontic adhesives included in the study.

Orthodontic Adhesive	Manufacturer	Components
Opal Seal	Opal Orthodontics, South Jordan, UT, USA	HPMA, Bis-GMA; glass ceramic and nanofillers, fluoride
Light Bond	Reliance Ortho Prod. Inc., Itasca, Il, USA	UDMA, TEGDMA; 85% fused silica, fluoride
Blugloo	Ormco Corporation, Glendora, CA, USA	Uncured methacrylate monomer; inert material fillers, fused silica, fluoride
Experimental material C1	“Raluca Ripan” Institue for Research in Chemistry, Cluj-Napoca, Romania	Bis-GMA, TEGDMA, UDMA; 80% Silanized strontium and zirconium fluoro-silicate glass, colloidal silica, quartz

HPMA: N-(2-Hydroxypropyl) methacrylamide (Sigma-Aldrich, Saint Louis, MO, USA); BIS-GMA: bisphenol A-glycidyl methacrylate (Sigma-Aldrich, Saint Louis, MO, USA); UDMA: urethane dimetacrylate (Sigma-Aldrich, Saint Louis, MO, USA).

**Table 2:** Values of shear bond strength of the four materials.

Material	Shear Bond Strength	(MPa)	*p*
	Mean	SD	
Opal Seal	8.086	1.507	<0.05 *
Light Bond	9.824	3.082	
Blugloo	8.898	1.459	
C1	9.052	1.741	

* statistically significant for two-way Anova test.

**Table 3:** Roughness values measured with AFM for the initial samples.

Sample	Scanned Zone					Average Value	*p*
	1	2	3	4	5		
	Ra, nm						
Blugloo	221	162	131	93.8	207	162.96	<0.05 *
Light Bond	18.7	10.1	11.8	7.04	15.8	12.68	2.67
Opal Seal	3.08	3.68	2.76	2.74	7.03	3.85	<0.05 *
C1	169	140	91.2	39.3	19.9	102.68	<0.05 *
	Rq, nm						
Blugloo	275	198	169	122	247	202.20	<0.05 *
Light Bond	24.6	13.6	14.4	9.04	21.3	16.58	0.988
Opal Seal	4.02	4.76	3.48	3.59	10.8	4.73	0.945
C1	221	176	130	76.8	29.3	126.62	<0.05 *

* statistically significant for Wilcoxon test.

**Table 4:** Roughness values measured with AFM for the samples after 60 days.

Sample	Scanned Zone					Average Value	*p*
	1	2	3	4	5		
	Ra, nm						
Blugloo	6.04	9.59	9.65	12.7	8.08	9.21	0.018 *
Light Bond	10.1	16.0	17.9	14.2	15.7	14.78	0.048 *
Opal Seal	9.07	10.6	12.9	9.51	10.6	10.53	1.563
C1	45.4	64.9	40.4	35.1	31.2	43.4	0.036 *
	Rq, nm						
Blugloo	7.38	11.8	12.6	15.8	10.3	11.57	0.008 *
Light Bond	12.5	19.6	21.5	18.1	19.7	18.28	0.040 *
Opal Seal	11.7	13.4	16.2	12.6	13.4	13.46	0.065
C1	58.5	82.1	50.4	43.2	38.7	54.58	0.001 *

* statistically significant for Wilcoxon test.

## Data Availability

Data sharing is not applicable.
